# A Linear Rehabilitative Motion Planning Method with a Multi-Posture Lower-Limb Rehabilitation Robot

**DOI:** 10.3390/s24237506

**Published:** 2024-11-25

**Authors:** Xincheng Wang, Musong Lin, Lingfeng Sang, Hongbo Wang, Yongfei Feng, Jianye Niu, Hongfei Yu, Bo Cheng

**Affiliations:** 1 Hebei Provincial Key Laboratory of Parallel Robot and Mechatronic System, Yanshan University, Qinhuangdao 066000, China; wangxincheng@stumail.ysu.edu.cn (X.W.); hongbo_w@ysu.edu.cn (H.W.); jyniu@ysu.edu.cn (J.N.); 2 School of Mechanical Engineering, Yanshan University, Qinhuangdao 066000, China; 3 Department of Environmental Engineering, Hebei University of Environmental Engineering, Qinhuangdao 066000, China; linmusong@hebuee.edu.cn; 4 Ningbo Key Laboratory of Aging Health Equipment and Service Technology, Ningbo Polytechnic, Ningbo 315211, China; sanglingfeng@163.com; 5 Academy for Engineering & Technology, Fudan University, Shanghai 200000, China; 6 Faculty of Mechanical Engineering & Mechanics, Ningbo University, Ningbo 315211, China; fengyongfei@nbu.edu.cn; 7 Collaborative Innovation Center for Port Industry Development in Coastal Areas, Qinhuangdao 066000, China; 8 Hebei Technology Innovation Center for Intelligent Industrial Design, Qinhuangdao 066000, China; 9 Qinhuangdao Hospital of Traditional Chinese Medicine, Qinhuangdao 066000, China

**Keywords:** rehabilitative motion planning, multi-posture lower-limb rehabilitation robot, joint rehabilitation, high-order polynomial curves, minimized jerk

## Abstract

In rehabilitation, physicians plan lower-limb exercises via linear guidance. Ensuring efficacy and safety, they design patient-specific paths, carefully plotting smooth trajectories to minimize jerks. Replicating their precision in robotics is a major challenge. This study introduces a linear rehabilitation motion planning method designed for physicians to use a multi-posture lower-limb rehabilitation robot, encompassing both path and trajectory planning. By subdividing the lower limb’s action space into four distinct training sections and classifying this space, we articulate the correlation between linear trajectories and key joint rehabilitation metrics. Building upon this foundation, a rehabilitative path generation system is developed, anchored in joint rehabilitation indicators. Subsequently, high-order polynomial curves are employed to mimic the smooth continuity of traditional rehabilitation trajectories and joint motions. Furthermore, trajectory planning is refined through the resolution of a constrained quadratic optimization problem, aiming to minimize the abrupt jerks in the trajectory. The optimized trajectories derived from our experiments are compared with randomly generated trajectories, demonstrating the suitability of trajectory optimization for real-time rehabilitation trajectory planning. Additionally, we compare trajectories generated based on the two groups of joint rehabilitation indicators, indicating that the proposed path generation system effectively assists clinicians in executing efficient and precise robot-assisted rehabilitation path planning.

## 1. Introduction

It is important to restore balance and walking ability for lower-limb rehabilitation in stroke. Li et al. [1,2,3] proposed that stroke leads to a decrease in knee joint flexion on the paralyzed side, and the knee flexion should be considered in therapy. Rybar et al. [4,5,6] emphasized the importance of hip flexors for standing and walking. The enhancement of lower-limb joint flexion ability helps to improve walking ability and gait speed in hemiplegic patients [7,8]. In addition, Schindler-Ivens et al. [9] pointed out that stroke patients have more passive joint extension, and additional joint extension exercise is unlikely to improve their motor ability. Pollock et al. [10] pointed out that training the affected lower limb in a rapid flexion mode may improve the walking and balance ability. Gomez-Cuaresma et al. [11] suggested that prolonged passive stretching of the entire motion range may help improve spasticity. In short, the ultimate joint flexion angle, the ultimate joint flexion frequency, and the ultimate joint motion range are important indicators of rehabilitation. Therefore, this paper explores the relationship between the linear trajectories and the corresponding joint rehabilitation characteristics of the indicators.

The passive training of linear trajectory is mainly achieved by physicians dragging the patient’s impaired limb or through the assistance of the rehabilitation robot. In traditional therapy, physicians can plan the optimal trajectories by touch and experience. However, when using the robot, physicians cannot learn the joints’ rehabilitation characteristics corresponding to the preset trajectory, making it difficult to plan the optimal trajectory. Typical multi-DOF lower-limb rehabilitation robots usually complete fixed trajectories through a single driving mechanism. The Lambda [12], the symmetrical lower-limb rehabilitation robot [13], and the CPM/CAM physiotherapy device [14] can complete circular trajectories. Horizontal lower limbs rehabilitation robot [15] and Fisiotek [16] can complete the horizontal linear trajectory. Due to their simple structure and low cost, these types of robots usually use a relatively simple trajectory planning method. Sitting and lying exoskeleton lower-limb rehabilitation robot is applicable to patients in multiple rehabilitation stages. The Motion Maker [17,18] can automatically guide patients to perform passive flexion training on the hip, knee, and ankle joints along a pre-selected trajectory. Other typical sitting and lying rehabilitation robots include Physiotherabot [19], TEMLX2 typeD [20], and NeXOS [21]. The sitting–lying lower-limb rehabilitation robot [22] provides a teaching training function for physicians, allowing them to plan personalized trajectories through the touch screen. Wearable exoskeleton robots and suspended rehabilitation robots are mainly used for gait rehabilitation, which are mainly composed of two symmetrical mechanical legs. Rewalk [23,24] can simulate the normal gait of the human body at an appropriate speed based on a preset motion model. Other typical wearable exoskeleton robots include HAL [25,26], Exo-H2 [27,28], and KineAssist [29]. Lokomat [30,31,32] is a suspended rehabilitation robot with a weight reduction suspension system. It guides motion based on preset gait motion patterns. Other typical suspended rehabilitation robots include LOPES [33] and ALEX [34]. The above gait rehabilitation robots either use predefined gait trajectories or use the mapping of healthy limbs to plan the trajectory. In addition, Guo et al. [35,36] designed a wearable teaching device for physicians to provide personalized gait trajectories for patients.

The above lower-limb rehabilitation robots have provided various trajectory planning methods for physicians. However, the preset gait trajectory is based on the trajectory of the function limb, which is not suitable for the sitting and lying robot [37]. The mapping of the function limb to the impaired limb requires auxiliary wearable equipment or structure with both legs, unfitting for robots with a single leg. The teaching device worn on the physician’s leg may result in a mismatch of joint mobility, leading to the trajectory exceeding a safe range. Moreover, it requires physicians relatively more labor and time. The preset or custom trajectory through the screen is common and easy to implement, but it is difficult for physicians to select or draw the optimal trajectory based on the rehabilitation needs of joints.

On the other hand, the previously reported works mostly focused on offline programming tasks [38,39]. With the increasing demands for rehabilitation, an increasing number of practical tasks require the rapid planning of movements for real-time execution. Therefore, real-time trajectory planning for rehabilitation robots has consistently been an important issue for generating safe and efficient trajectories. Real-time trajectory planning has been well applied in the field of autonomous driving. However, in dynamic environments, real-time trajectory planning based on optimization that is specifically tailored to the joint rehabilitation needs of rehabilitation robots is rarely found in the literature. The reported real-time trajectory planning in the references is limited to path generation and does not address the performance of the robot and the rehabilitation.

A multistage hemiplegic lower-limb rehabilitation robot (MHLRR) [40] was designed by our team to conduct motor rehabilitation of lower limbs for patients with hemiplegia in multiple recovery stages. It has several features, including multistage usability, bi-side usability, lightweight design, mechanically adjustable limits, and easy mobility. Based on the lower-limb rehabilitation robot, this paper will deal with the foregoing problem, which is structured as follows:

- Step 1: Patient-specific lower-limb parameters are input into the system, leading to the creation of a personalized lower-limb motion space. This space is subsequently segmented into distinct training regions, categorized according to the classification criteria established for the space. Building upon this classification, the initial and final positions, as well as intermediate via points, are meticulously defined. These points are strategically positioned within the valid training region of the action space, aligning with the five joint rehabilitation indicator parameters that have been set by a physician based on their clinical experience.
- Step 2: Utilizing the defined key points, a parametric path is constructed via a seventh-degree polynomial curve. The coefficients of this polynomial are then fine-tuned through optimization to accurately represent the intended rehabilitation trajectory. The robot’s end effector is designed to minimize jerk, replicating a gentle and low-impact rehabilitative trajectory. This mimics the approach traditionally employed by physicians, who guide the affected limbs along such trajectories during therapy.
- Step 3: The kinematic curve of the rehabilitation trajectory is converted into joint space through inverse kinematics. The joint movements are then transmitted to the controller, which drives the lower-limb rehabilitation robot’s end effector to track the generated trajectory.

The remainder of this paper is organized as follows. Section 2 introduces the multi-posture lower-limb rehabilitation robot. Section 3 presents the rehabilitative path planning based on the joint rehabilitation indicators. In Section 4, the rehabilitative trajectory planning-based jerk minimization is illustrated with the two experimental rehabilitative trajectories to verify the effectiveness. Finally, the conclusions are drawn.

## 2. Multi-Posture Lower-Limb Rehabilitation Robot

### 2.1. Mechanism

**Figure 1:**
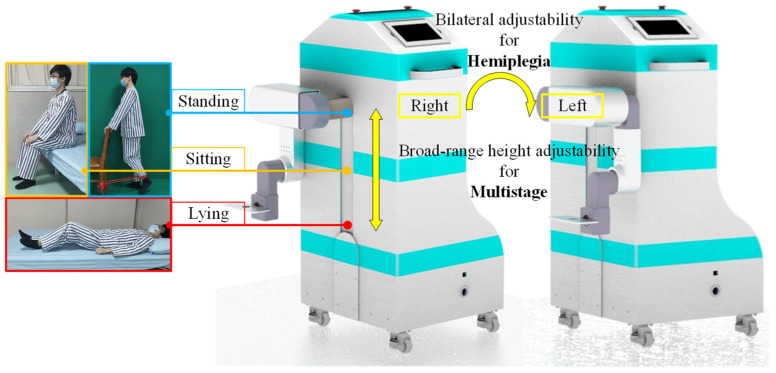
Prototype of the MHLRR. MHLRR: Multistage Hemiplegic Lower-Limb Rehabilitation Robot. Multiple training postures for patients in all stages of recovery. Multiple training sides for patients with hemiplegia.

As shown in Figure 1, the primary design considerations are the adaptability of rehabilitation in multiple recovery stages and the adjustability on the bilateral training side. For multistage rehabilitation based on Brunnstrom’s theory, the MHLRR’s training posture must be versatile enough to support patients in lying, sitting, and standing positions, aligning with their stages of recovery. Furthermore, the robot’s training apparatus should be configurable to address the specific hemiplegic side, ensuring personalized therapy. To this end, it is vital that the ranges of joint motion and the height of leg orthosis can be adjusted according to the recovery stage and the affected side.

**Figure 2:**
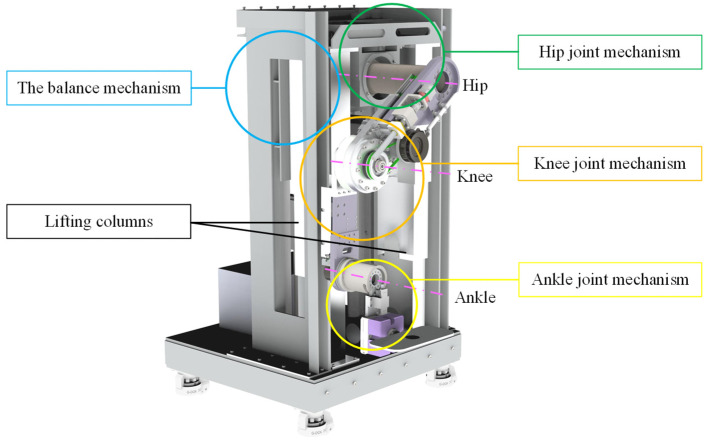
Overall structure of the MHLRR.

Based on the modular design concept, the MHLRR mainly consists of four parts, including the balance mechanism, the hip joint mechanism, the knee joint mechanism, and the ankle joint mechanism, as depicted in Figure 2. This design philosophy ensures that the leg orthosis can be adjusted in height from 540 mm to 1040 mm, providing a versatile range that spans from the hip joint axis to the ground level. This adaptability is achieved through the manipulation of the lifting columns, catering to the diverse needs of patients across various stages of recovery and enabling rehabilitation in lying, sitting, or standing positions. This is achieved by actuating the hip joint motor, which symmetrically aligns the leg orthosis with respect to the coronal plane, thus allowing for bilateral training adaptability.

### 2.2. Description of the Action Space

**Figure 3:**
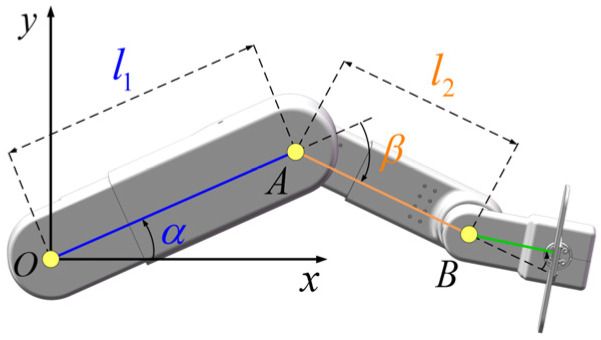
The kinematic model.

According to the physiological structure of the human lower limb, the rehabilitation robot is simplified to a link structure, as shown in Figure 3. The dimensions of the thigh and calf are denoted as l1 and l2, respectively. The kinematic model is depicted in Figure 3. The symbols *O*, *A*, and *B* correspond to the hip joint, knee joint, and ankle joint, respectively. The rotation angles of the hip and knee joints are denoted by *α* and *β*, respectively. The hip joint’s center, point *O*, is established as the coordinate system’s origin, with the ankle joint B designated as the terminal point. Subsequently, the relationship between the coordinates of point B and the joint angles can be derived using forward and inverse kinematics as follows:

(1) $$\left\{\begin{array}{l} x_{B}=l_{1}\cdot \cos\alpha +l_{2}\cdot \cos(\alpha +\beta )\\ y_{B}=l_{1}\cdot \sin\alpha +l_{2}\cdot \sin(\alpha +\beta )\\ \alpha =\arctan\frac{y_{B}}{x_{B}}+\arccos\frac{{l_{1}}^{2}-{l_{2}}^{2}+{x_{B}}^{2}+{y_{B}}^{2}}{2\cdot l_{1}\cdot \sqrt{{x_{B}}^{2}+{y_{B}}^{2}}}\\ \beta =\arccos\frac{{x_{B}}^{2}+{y_{B}}^{2}-{l_{1}}^{2}-{l_{2}}^{2}}{2\cdot l_{1}\cdot l_{2}} \end{array}\right.$$

**Figure 4:**
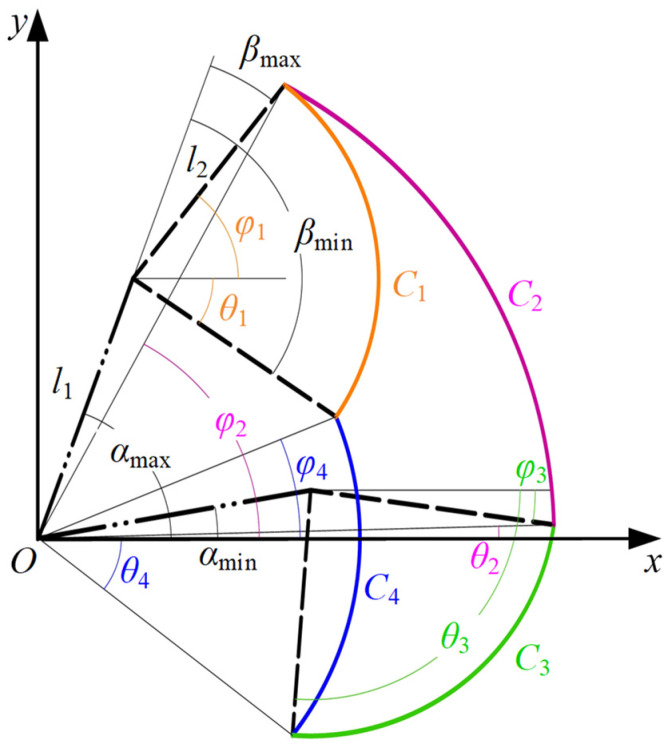
Definition and nomenclature of the action space. This figure represents a sitting position, with the patient’s joint ranges of motion set to *α*_max_ = 75°, *α*_min_ = 10°, *β*_max_ = −5°, and *β*_min_ = −95°.

The workspace of the proposed robot is adjustable according to the different action spaces of patients. It is determined by four factors, including the affected side, the training posture, the joint motion range, and the lower-limb length. Figure 4 shows the definition of the action space of a common patient in a sitting position.

Point *O* signifies the hip joint, while *l*_1_ and *l*_2_ denote the lengths of the thigh and calf, respectively. The terms *α*_min_ and *α*_max_ correspond to the minimum and maximum flexion angles of the hip joint, respectively, while *β*_min_ and *β*_max_ denote the minimum and maximum extension angles of the knee joint. In our coordinate system, counterclockwise rotation is designated as the positive direction. The hip angle is conventionally set to zero in both sitting and lying positions when the thigh is aligned parallel to the horizontal axis. In parallel, the knee angle is also defined as zero when the calf is in direct alignment with the thigh, irrespective of the patient’s position. This standardized approach ensures consistency and clarity in the measurement and analysis of joint angles throughout the rehabilitation process. Curve *C*_1_ delineates the path of the end point as the knee joint moves from its minimum to maximum angle with the hip joint fixed at its maximum angle. Similarly, the three other curves, *C*_2_, *C*_3,_ and *C*_4,_ can be obtained. To enhance the clarity of these four workspace curves, we introduce Equation (2) for a more comprehensive depiction, which is expressed as

(2) $$Ci=(x_{i},y_{i},r_{i},\theta_{i},\varphi_{i})(i=1,2,3,4),$$

where *θ_i_*, *φ_i_*, (*x_i_*, *y_i_*), and *r_i_* represent the start angle, the end angle, the center coordinates, and the radius of the curve *C_i_*, respectively. Then, the expressions for each parameter can be obtained as

(3) $$\left\{\begin{array}{l} r_{1}=l_{2};r_{2}=\sqrt{{l_{1}}^{2}+{l_{2}}^{2}+2\cdot l_{1}\cdot l_{2}\cdot \cos\beta \max}\\ r_{3}=l_{2};r_{4}=\sqrt{{l_{1}}^{2}+{l_{2}}^{2}+2\cdot l_{1}\cdot l_{2}\cdot \cos\beta \min}\\ \left(x_{1},y_{1})=(l_{1}\cdot \cos\alpha \max,l_{1}\cdot \sin\alpha \max);(x_{2},y_{2})=(0,0\right)\\ \left(x_{3},y_{3})=(l_{1}\cdot \cos\alpha \min,l_{1}\cdot \sin\alpha \min);(x_{4},y_{4})=(0,0\right)\\ \theta_{1}=\alpha \max+\beta \min;\theta_{2}=\alpha \min-\arccos\frac{l_{1}+l_{2}\cdot \cos\beta \max}{\sqrt{{l_{1}}^{2}+{l_{2}}^{2}+2\cdot l_{1}\cdot l_{2}\cdot \cos\beta \max}}\\ \theta_{3}=\alpha \min+\beta \min;\theta_{4}=\alpha \min-\arccos\frac{l_{1}+l_{2}\cdot \cos\beta \min}{\sqrt{{l_{1}}^{2}+{l_{2}}^{2}+2\cdot l_{1}\cdot l_{2}\cdot \cos\beta \min}}\\ \varphi_{1}=\alpha \max+\beta \max;\varphi_{2}=\alpha \max-\arccos\frac{l_{1}+l_{2}\cdot \cos\beta \max}{\sqrt{{l_{1}}^{2}+{l_{2}}^{2}+2\cdot l_{1}\cdot l_{2}\cdot \cos\beta \max}}\\ \varphi_{3}=\alpha \min+\beta \max;\varphi_{4}=\alpha \max-\arccos\frac{l_{1}+l_{2}\cdot \cos\beta \min}{\sqrt{{l_{1}}^{2}+{l_{2}}^{2}+2\cdot l_{1}\cdot l_{2}\cdot \cos\beta \min}} \end{array}\right..$$

It serves as the theoretical foundation for calculating the coordinates of the starting and ending points of the linear trajectory when it is at different positions within the action space.

The maximum joint ranges of motion of MHLRR under lying, sitting, and standing postures are shown in Table 1.

## 3. Rehabilitative Path Planning Based on Joint Rehabilitation Indicators

### 3.1. The Division of Training Section and the Classification of the Action Space

The CPM (Continuous Passive Motion) linear trajectory training is a widely adopted rehabilitation technique, typically implemented by clinicians who manually guide the patient’s lower limb through a reciprocating linear motion. This method is particularly beneficial during the early recovery stages for patients experiencing muscle paralysis or diminished muscle strength, as it facilitates passive training that preserves the joint’s large range of motion, thereby preventing joint contractures and deformities. As patients progress into the middle and late stages of recovery, where they may have regained some muscle strength but still lack the ability to achieve full joint flexion, a more targeted approach becomes necessary. This involves focusing on maximum flexion training to further enhance joint mobility.

Building on these principles, this study delves into the joint rehabilitation characteristics of the CPM linear trajectory in both lying and sitting positions. The analysis aims to tailor the rehabilitation process to patients with diverse action spaces and varying joint rehabilitation requirements, ensuring a more personalized and effective treatment plan.

To effectively encapsulate the joint rehabilitation characteristics of the horizontal linear trajectory ensemble within the dynamic action space, it is imperative to quantify the distinct types of lower-limb action spaces. The variables *P_ij_* (*i* = 1, 3; *j* = 2, 4) are designated as the intersection points between arcs *C_i_* and *C_j_*. Points *M* and *N* represent the initiation and termination points, respectively, of the intersection between the horizontal linear trajectory and the action space. Points *Q*_1_ and *Q*_2_ are identified as the upper tangent points of the circle encompassing arcs *C*_1_ and *C*_2_, while *Q*_3_ and *Q*_4_ correspond to the lower tangent points of the circle for arcs *C*_3_ and *C*_4_. The coordinates of these eight pivotal points can be ascertained through kinematic analysis as follows:

(4) $$\left\{\begin{array}{l} P_{12}(l_{1}\cdot \cos\alpha_{\max}+l_{2}\cdot \cos(\alpha_{\max}+\beta_{\max}),l_{1}\cdot \sin\alpha_{\max}+l_{2}\cdot \sin(\alpha_{\max}+\beta_{\max}))\\ P_{14}(l_{1}\cdot \cos\alpha_{\max}+l_{2}\cdot \cos(\alpha_{\max}+\beta_{\min}),l_{1}\cdot \sin\alpha_{\max}+l_{2}\cdot \sin(\alpha_{\max}+\beta_{\min}))\\ P_{23}(l_{1}\cdot \cos\alpha_{\min}+l_{2}\cdot \cos(\alpha_{\min}+\beta_{\max}),l_{1}\cdot \sin\alpha_{\min}+l_{2}\cdot \sin(\alpha_{\min}+\beta_{\max}))\\ P_{34}(l_{1}\cdot \cos\alpha_{\min}+l_{2}\cdot \cos(\alpha_{\min}+\beta_{\min}),l_{1}\cdot \sin\alpha_{\min}+l_{2}\cdot \sin(\alpha_{\min}+\beta_{\min}))\\ Q_{1}(l_{1}\cdot \cos\alpha_{\max},l_{1}\cdot \sin\alpha_{\max}+l_{2});Q_{2}(0,\sqrt{{l_{1}}^{2}+{l_{2}}^{2}+2\cdot l_{1}\cdot l_{2}\cdot \cos\beta_{\max}})\\ Q_{3}(l_{1}\cdot \cos\alpha_{\min},l_{1}\cdot \sin\alpha_{\min}-l_{2});Q_{4}(0,-\sqrt{{l_{1}}^{2}+{l_{2}}^{2}+2\cdot l_{1}\cdot l_{2}\cdot \cos\beta_{\min}}) \end{array}\right..$$

The action space is segmented into multiple training sections by horizontal lines intersecting at these key points. The principles for section division are as follows: within the same section, all starting points of linear trajectories are on the same arc *C_i_*. Moreover, all ending points are also on the same arc *C_j_*. Across different sections, there exists at least one pair of trajectories whose starting or ending points do not align on the same arc. Additionally, sections composed of identical arcs *C_i_C_j_* (where *i* = *j*) are designated as section 0, while those composed of distinct arcs *C_i_C_j_* (where *i* ≠ *j*) are sequentially labeled as sections 1 to 3 from the bottom upward.

**Figure 5:**
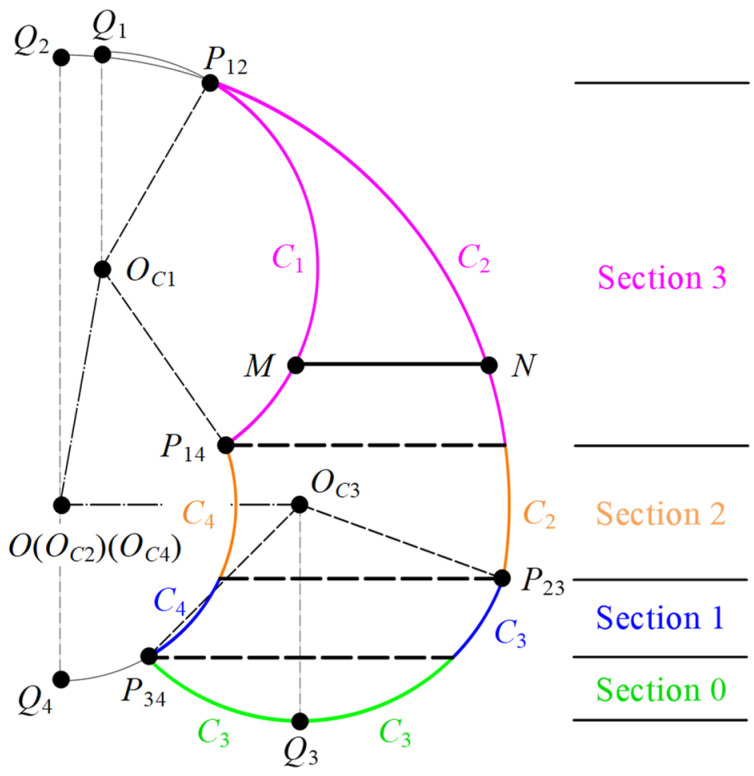
Section division of the action space.

Each training section is demarcated by boundary lines intersecting at key points and a pair of arcs, as illustrated in Figure 5. Points *P*_12_, *Q*_1_, and *Q*_2_ are situated above *P*_14_ and *P*_23_, whereas points *P*_34_, *Q*_3_, and *Q*_4_ are positioned below *P*_14_ and *P*_23_, according to the graphical method. Consequently, the action space is stratified into three distinct regions: the bottom, the middle, and the top.

The classification method of the action space is proposed by analyzing the distribution of key points across the bottom, middle, and top regions. Firstly, we focus on the positioning of points *P*_34_, *Q*_3_, and *Q*_4_ within the bottom region of the action space. The scenarios for one situation in the lying position and two in the sitting position are depicted in Figure 5. It is crucial to recognize that in stroke patients with lower-limb impairments, the hip joint’s free extension is typically unimpaired, whereas achieving a significant flexion angle is more challenging. Consequently, the minimum flexion angles for the hip joint in the lying and sitting positions are established at *α*_min_ = 50° and *α*_min_ = 0°, respectively.

**Figure 6:**
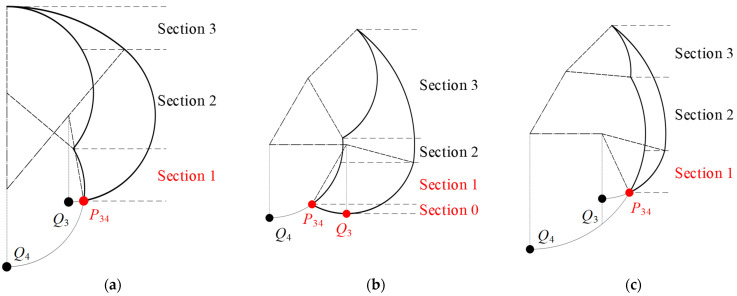
The positioning of points P34, Q3, and Q4 within the bottom region of the action space: (**a**) *x_Q_*_4_ < *x_Q_*_3_ < *x_P_*_34_ in lying position; (**b**) *x_Q_*_4_ < *x_P_*_34_ < *x_Q_*_3_ in sitting position; (**c**) *x_Q_*_4_ < *x_Q_*_3_ ≤ *x_P_*_34_ in sitting position.

Utilizing Equation (4), it becomes evident that the positions of the three points *P*_34_, *Q*_3_, and *Q*_4_ are interrelated with *α*_min_, *β*_min_, *l*_1,_ and *l*_2_. In the lying position, there is a singular scenario where *x_Q_*_4_ < *x_Q_*_3_ < *x_P_*_34_, given the conditions *α*_min_ = 50°, −135° ≤ *β*_min_ < 0° and Equation (5). This leads to the conclusion that in the lying position, there is a single configuration at the bottom of the action space: when −135° ≤ *β*_min_ < 0°, *P*_34_ is the lowest point, and both *Q*_3_ and *Q*_4_ lie outside the action space, as shown in Figure 6a.

In the sitting position, the condition *x_Q_*_4_ < *x_P_*_34_ can be deduced from *α*_min_ = 0°, −135° ≤ *β*_min_ < 0°, and *l*_1_ > *l*_2_. Subsequently, based on Equation (4), it can be obtained that −135° ≤ *β*_min_ < −90° when *x_Q_*_4_ < *x_P_*_34_ < *x_Q_*_3_, and −90° ≤ *β*_min_ ≤ 0° when *x_Q_*_4_ < *x_Q_*_3_ ≤ *x_P_*_34_. Therefore, it can be concluded that in the sitting position, there are two distinct scenarios at the bottom of the action space. When −135° ≤ *β*_min_ < −90°, *P*_34_ serves as the critical boundary point, *Q*_3_ is the lowest and critical boundary point, and *Q*_4_ is outside the action space, as shown in Figure 6b. Conversely, when −90° ≤ *β*_min_ ≤ 0°, *P*_34_ is both the lowest and critical boundary point, with *Q*_3_ and *Q*_4_ situated outside the action space, as shown in Figure 6c.

**Figure 7:**
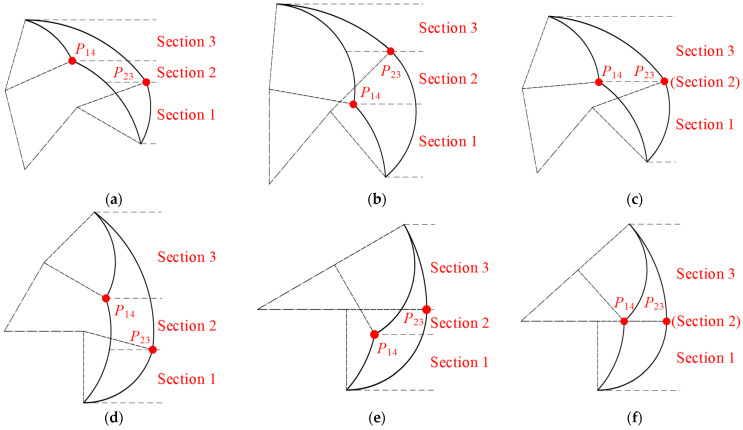
The positioning of points *P*_14_ and *P*_23_ within the middle region of the action space: (**a**) *y_P_*_14_ > *y_P_*_23_ in lying position; (**b**) *y_P_*_14_ < *y_P_*_23_ in lying position; (**c**) *y_P_*_14_ = *y_P_*_23_ in lying position; (**d**) *y_P_*_14_ > *y_P_*_23_ in sitting position; (**e**) *y_P_*_14_ < *y_P_*_23_ in sitting position; (**f**) *y_P_*_14_ = *y_P_*_23_ in sitting position.

Secondly, we analyze the positioning of points *P*_14_ and *P*_23_ within the middle region of the action space, which encompasses six distinct scenarios: *y_P_*_14_ > *y_P_*_23_, *y_P_*_14_ < *y_P_*_23_, and *y_P_*_14_ = *y_P_*_23_ in lying and sitting positions. According to Equation (4), the positional relationship between these two points is influenced by all the parameters involved. By solving for the conditions that satisfy each of these scenarios, we can determine their relative positions, as illustrated in Figure 7.

Consequently, we can deduce the following conclusions: Point *P*_14_ is situated above *P*_23_ when the inequality *l*_1_·sin*α*_max_ – *l*_1_·sin*α*_min_ + *l*_2_·sin(*α*_max_ + *β*_min_) – *l*_2_·sin(*α*_min_ + *β*_max_) > 0 holds true. Conversely, *P*_14_ is located below *P*_23_ when the inequality *l*_1_·sin*α*_max_ – *l*_1_·sin*α*_min_ + *l*_2_·sin(*α*_max_ + *β*_min_) – *l*_2_·sin(*α*_min_ + *β*_max_) < 0 is met. Lastly, points *P*_14_ and *P*_23_ are aligned at the same height when the equation *l*_1_·sin*α*_max_ – *l*_1_·sin*α*_min_ + *l*_2_·sin(*α*_max_ + *β*_min_) −*l*_2_·sin(*α*_min_ + *β*_max_) = 0 is satisfied.

**Figure 8:**
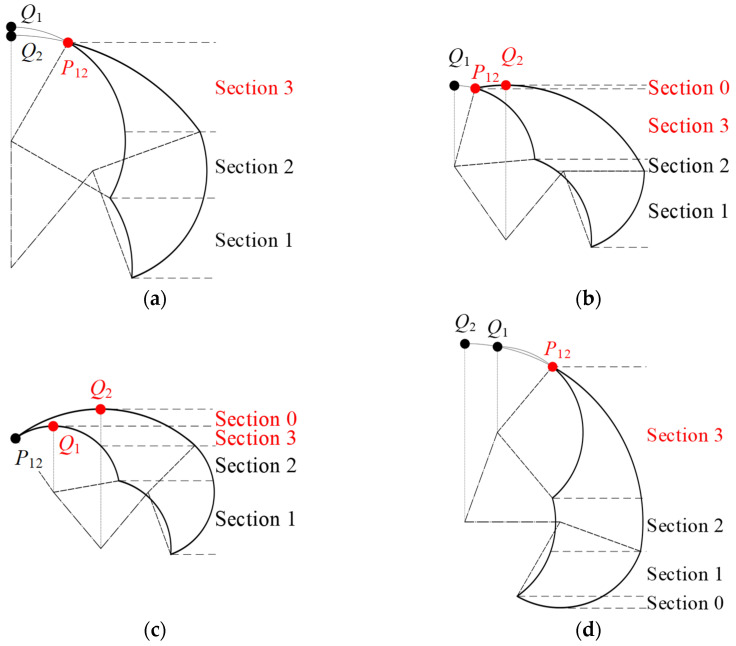
The positioning of points *P*_12_, *Q*_1_, and *Q*_2_ within the top region of the action space: (**a**) *x_Q_*_1_ ≤ *x_P_*_12_ and *x_Q_*_2_ ≤ *x_P_*_12_ in a lying position; (**b**) *x_Q_*_1_ ≤ *x_P_*_12_ < *x_Q_*_2_ in a lying position; (**c**) *x_P_*_12_ < *x_Q_*_1_ < *x_Q_*_2_ in a lying position; (**d**) *x_Q_*_2_ < *x_Q_*_1_ < *x_P_*_12_ in sitting position.

Thirdly, proceeding to the analysis of points *P*_12_, *Q*_1_, and *Q*_2_ in the top region of the action space, we observe three distinct scenarios in the lying position and a single scenario in the sitting position, as depicted in Figure 8. It is important to note that patients with stroke-induced lower-limb disorders often have limited knee joint flexion, while the knee’s free extension is typically preserved. Hence, the knee joint’s maximum angle range in both lying and sitting positions is confined to −90° < *β*_max_ ≤ 0°.

Equation (5) reveals that the positions of these three points are interdependent on *α*_max_, *β*_max_, *l*_1,_ and *l*_2_. In the lying position, we first examine the relationship between *x_Q_*_1_ and *x_Q_*_2_. When *x_Q_*_1_ ≥ *x_Q_*_2_, there is a singular scenario where *x_Q_*_2_ ≤ *x_Q_*_1_ ≤ *x_P_*_12_, which corresponds to the condition 50° < *α*_max_ ≤ 90°. When *x_Q_*_1_ < *x_Q_*_2_, three possible situations arise: *x_Q_*_1_ < *x_Q_*_2_ ≤ *x_P_*_12_, *x_Q_*_1_ ≤ *x_P_*_12_ < *x_Q_*_2_, and *x_P_*_12_ < *x_Q_*_1_ < *x_Q_*_2_, each with specific conditions that can be solved. Thus, in the lying position, there are three possible configurations at the top of the action space. For 50° < *α*_max_ ≤ 90°, or when 90° < *α*_max_ ≤ 130° and *β*_max_ ≥ 180° − *α*_max_ − arccos(cos*α*_max_·*l*_1_/*l*_2_), there exists *x_Q_*_1_ ≤ *x_P_*_12_ and *x_Q_*_2_ ≤ *x_P_*_12_. Here, *P*_12_ is the highest and critical boundary point, with both *Q*_1_ and *Q*_2_ either outside or coinciding with the action space. For 90° < *α*_max_ ≤ 130°, *β*_max_ < 180° − *α*_max_ − arccos(cos*α*_max_·*l*_1_/*l*_2_), and −*α*_max_ + 90° ≤ *β*_max_ ≤ −*α*_max_ + 130°, there exists *x_Q_*_1_ ≤ *x_P_*_12_ < *x_Q_*_2_. In this case, *Q*_2_ is the highest and critical boundary point, *P*_12_ is a critical boundary point, and *Q*_1_ is outside the action space. When 90° < *α*_max_ ≤ 130°, *β*_max_ < 180° − *α*_max_ − arccos(cos*α*_max_·*l*_1_/*l*_2_), and −*α*_max_ + 90° ≤ *β*_max_ ≤ −*α*_max_ + 130°, there exists *x_P_*_12_ < *x_Q_*_1_ < *x_Q_*_2_. Here, *Q*_2_ is the highest and critical boundary point, *Q*_1_ is a critical boundary point, and *P*_12_ is merely a common boundary point.

In the sitting position, given the conditions 0° < *α*_max_ ≤ 80° and −90° < *β*_max_ ≤ 0°, it is determined that *x_Q_*_2_ < *x_Q_*_1_ < *x_P_*_12_. Therefore, there is one singular scenario at the top of the action space in the sitting position, where *P*_12_ is the highest and critical boundary point, and both *Q*_1_ and *Q*_2_ are situated outside the action space.

Following the comprehensive analysis of the key boundary points across the bottom, middle, and top regions, we can conclude that there are nine situations in a lying position and six situations in a sitting position. Building on this foundation, a classification method for the action space is devised, where the action spaces with the same section number and one-to-one correspondence of key boundary points are considered of the same type.

There are three pairs of scenarios in the lying and sitting positions that are classified as the same type. Specifically, the combinations of Figure 6a, Figure 7a and Figure 8a, Figure 6a, Figure 7b and Figure 8a, and Figure 6a, Figure 7c and Figure 8a in the lying position correspond to the same type as Figure 6c, Figure 7d and Figure 8d, Figure 6c, Figure 7e and Figure 8d, and Figure 6c, Figure 7f and Figure 8d in the sitting position, respectively. Consequently, the action space is composed of 12 distinct types based on the section division principle, as outlined in Table 2.

It is important to highlight that Types 1~3 have three conditions (a, b, and c) and are associated with two training postures (lying and sitting), whereas Types 4~12 are characterized by a single condition and a single training posture. Most significantly, within the action space of the same type, the group of linear trajectories exhibits identical rehabilitation characteristics and patterns for both the hip and knee joints, ensuring a consistent approach to rehabilitation across various patients in the same scenarios.

### 3.2. The Characteristics of Joint Rehabilitation Corresponding to the Rehabilitative Path

Points *M* and *N* are designated as the origin and terminus, respectively, of the linear trajectory L. The angles *α_L_*_max_ and *α_L_*_min_ represent the maximum and minimum hip flexion angles along trajectory L, while *β_L_*_max_ and *β_L_*_min_ denote the corresponding maximum and minimum knee extension angles. Furthermore, *α_M_* and *β_M_* are the hip and knee joint angles at the ankle’s position *M*, and *α_N_* and *β_N_* are the respective angles at position *N*. The midpoint *T* of trajectory *L* is accompanied by angles *α_T_* and *β_T_*, which are the hip and knee joint angles when the ankle reaches *T*.

**Figure 9:**
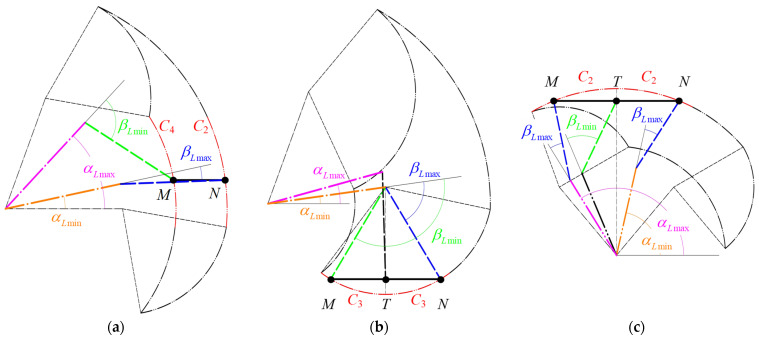
The positioning of the origin point *M* and the terminus point *N* of the linear trajectory: (**a**) *M* and *N* are on different arcs *C_i_C_j_* (with *i* ≠ *j*); (**b**) *M* and *N* are on the same arc *C*_3_*C*_3_; (**c**) *M* and *N* are on the same arc *C*_2_*C*_2_.

In the scenario where points *M* and *N* are located on different arcs *C_i_C_j_* (with *i* ≠ *j*), as depicted in Figure 9a, point *M* is aligned with the maximum flexion angles for both the hip and knee joints, whereas point *N* corresponds to their minimum flexion angles. When *M* and *N* are situated on the same arc *C*_3_*C*_3_, point *T* represents the hip joint at its maximum flexion angle, with *M* and *N* indicating the minimum hip flexion angle. Concurrently, *M* and *N* correspond to the minimum and maximum knee extension angles, respectively, as shown in Figure 9b.

In another configuration where *M* and *N* are on the same arc *C*_2_*C*_2_, *M* and *N* are associated with the maximum and minimum hip flexion angles, respectively. M and N also correspond to the maximum knee extension angle, while point *T* is at the minimum knee extension angle, as illustrated in Figure 9c. The limiting conditions for the joint angles corresponding to the linear trajectory in these three cases can be articulated as follows:

(5) $$\left\{\begin{array}{l} \alpha_{L\max}=\alpha_{M};\alpha_{L\min}=\alpha_{N};\beta_{L\max}=\beta_{N};\beta_{L\min}=\beta_{M};(C_{i}C_{j},i\neq j)\\ \alpha_{L\max}=\alpha_{T};\alpha_{L\min}=\alpha_{M}=\alpha_{N};\beta_{L\max}=\beta_{N};\beta_{L\min}=\beta_{M};(C_{i}C_{j},i=j=3)\\ \alpha_{L\max}=\alpha_{M};\alpha_{L\min}=\alpha_{N};\beta_{L\max}=\beta_{M}=\beta_{N};\beta_{L\min}=\beta_{T};(C_{i}C_{j},i=j=2) \end{array}\right.$$

For a detailed analysis of the joint rehabilitation features and patterns within various action spaces for the linear trajectory groups, Type 10 has been discussed. This targeted study aims to offer physicians a comprehensive guide and theoretical underpinning for the effective utilization of rehabilitation robots in their practice.

Moreover, drawing from the analytical findings of the selected types, we have crafted a comprehensive summary of the joint rehabilitation characteristics and principles applicable to the remaining types. This compilation aims to serve as a reference for healthcare professionals, enhancing their ability to tailor joint rehabilitation protocols to the specific needs of individual patients.

**Figure 10:**
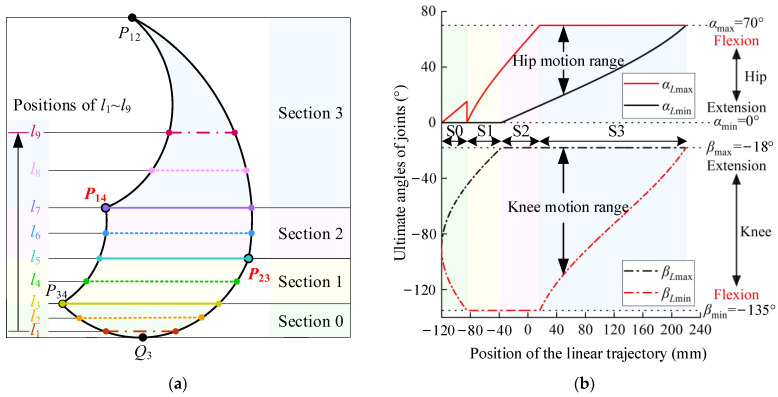
The positions and the ultimate joint angles of the linear trajectories within the action space of Type 10: (**a**) the nine linear trajectories (*l*_1_~*l*_9_) from bottom to top; (**b**) the ultimate angles of joints corresponding to the position of the linear trajectories.

Let us consider Type 10 as a case study. We have configured the hip joint’s motion range to span from 0° to 70° and the knee joint’s motion range from −135° to −18°. Additionally, the lengths of the thigh and calf have been standardized at 400 mm and 360 mm, respectively. With these parameters, the action space is graphically represented in Figure 10a, which is segmented into four distinct regions (green, yellow, pink, and blue), setting nine exemplary linear trajectories, *l*_1_ through *l*_9_, arranged vertically from bottom to top. An analysis of the joint rehabilitation characteristics corresponding to these trajectories follows.

The interplay between the linear trajectory’s position (*y_l_*) and the maximum flexion–extension angles of the hip and knee joints is depicted in Figure 10b. The hip joint’s position is established as the coordinate origin, with the horizontal axis denoting the trajectory’s height and the vertical axis representing the joint angles. As delineated in Equation (7), the linear trajectory will correspond to the hip joint’s maximum flexion motion when *α_L_*_max_ = *α*_max_. Similarly, the linear trajectory will correspond to the knee joint’s maximum flexion motion when *β_L_*_min_ = *β*_min_. An ultimate joint motion range is achieved when the maximum motion range of the patient’s joints matches that of the linear trajectory.

(6) $$\left\{\begin{array}{c} \alpha_{\min}\leq \alpha_{L\min}\leq \alpha_{L\max}\leq \alpha_{\max}\\ \beta_{\min}\leq \beta_{L\min}\leq \beta_{L\max}\leq \beta_{\max} \end{array}\right.$$

In the early stages of recovery, the emphasis of rehabilitation is placed on regaining muscle strength, making it crucial to engage in exercises that involve a wide range of joint motion. As patients progress into the middle or late stages of recovery, the focus shifts toward ensuring the joints can extend freely. At this point, the ultimate flexion training of the joints becomes particularly significant to enhance their functional capabilities and restore full mobility. As shown in Figure 10b, we analyze the influence of the linear trajectory’s section and position on the characteristics of joint rehabilitation.

As shown in Figure 10b, it is evident that the trajectory within section 0 (denoted by the green area) lacks both the broad joint motion range and the capacity for ultimate joint flexion. Consequently, section 0 is not an appropriate choice for planning linear trajectories aimed at joint rehabilitation training.

Moving on to section 1 (the yellow area), as the trajectory ascends, there is a gradual expansion in the joints’ range of motion. Notably, the knee joint maintains its ultimate flexion (*β_L_*_min_ = *β*_min_). Thus, the linear trajectories in section 1 are best suited for rehabilitation that emphasizes the knee joint as the primary focus, with the hip joint serving as a supportive element. In summary, the trajectories in section 1 preserve the knee joint’s ultimate flexion, and as the trajectory’s position rises, the intensity of joint training correspondingly increases.

Moving on to section 2 (the pink area), the trajectory’s upward movement is accompanied by the knee joint maintaining both ultimate flexion and maximum range of motion (*β_L_*_max_ = *β*_max_ and *β_L_*_min_ = *β*_min_). Additionally, the hip joint’s flexion angle and motion range increase progressively. Therefore, the linear trajectories in section 2 continue to prioritize the knee joint for rehabilitation, with the hip joint in a supportive role. In conclusion, the trajectories in section 2 sustain the knee joint’s ultimate flexion and maximum motion range, and the higher the trajectory’s position, the more intense the joint training becomes.

Lastly, in section 3 (the blue area), the trajectory’s ascent is characterized by the hip joint maintaining ultimate flexion (*α_L_*_max_ = *α*_max_) and a diminishing motion range. The knee joint’s motion range also decreases, and its ultimate flexion capability is lost. Moreover, the length of the linear trajectory is reduced. Under equivalent training conditions of speed and duration, the frequency of the hip joint’s ultimate flexion increases, enhancing the efficiency of the training of the hip joint. Thus, the linear trajectories in section 3 are designed with the hip joint as the primary focus and the knee joint as a supportive element. In conclusion, the higher the trajectory’s position, the more effective the training efficiency for the hip joint’s ultimate flexion.

**Figure 11:**
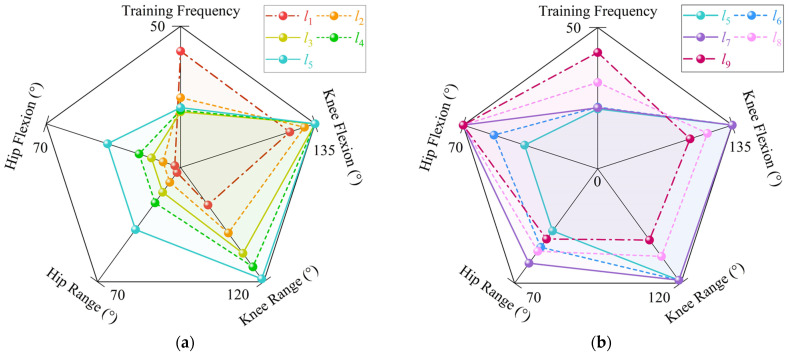
The five key metrics to evaluate joint rehabilitation of linear trajectories: (**a**) the key metrics of joint rehabilitation of *l*_1_~*l*_5_; (**b**) the key metrics of joint rehabilitation of *l*_5_~*l*_9_.

We have identified five key metrics to evaluate the joint rehabilitation effectiveness of the nine linear trajectories (*l*_1_~*l*_9_), as depicted in Figure 11a,b. These metrics include the ultimate hip joint flexion angle, the ultimate knee joint flexion angle, the maximum hip joint motion range, the maximum knee joint motion range, and the ultimate joint flexion frequency.

Specifically, linear trajectories *l*_1_ and *l*_2_ do not encompass the joints’ maximum motion range or ultimate flexion. Trajectories *l*_3_ and *l*_4_ are characterized by the inclusion of the knee joint’s ultimate flexion only. Trajectories *l*_5_ and *l*_6_ extend to include both the knee joint’s ultimate flexion and its maximum motion range. Trajectory *l*_7_ is distinguished by encompassing the knee joint’s ultimate flexion and maximum motion range, as well as the hip joint’s ultimate flexion. Furthermore, the training frequency for trajectories *l*_1_ through *l*_7_ is essentially uniform. Trajectories *l*_8_ and *l*_9_ exclusively feature the hip joint’s ultimate flexion, with *l*_9_ exhibiting the highest training frequency among all trajectories, indicating that, under identical training conditions, *l*_9_ achieves the greatest number of hip joint flexions.

It can be concluded that trajectory *l*_7_ is the optimal choice for comprehensive joint rehabilitation, as it includes the knee joint’s ultimate flexion, maximum motion range, and the hip joint’s ultimate flexion. Trajectories *l*_8_ and *l*_9_ are designed for enhanced efficiency in hip joint ultimate flexion. Trajectories *l*_5_ and *l*_6_, which include both the knee joint’s ultimate flexion and maximum motion range, offer a more intensive training focus on the hip joint. Trajectories *l*_3_ and *l*_4_, focusing solely on the knee joint’s ultimate flexion, provide a lower intensity of training for the hip joint. Lastly, trajectories *l*_1_ and *l*_2_ have minimal rehabilitative impact on the joints.

Following the analytical approach applied to Type 10, each of the remaining types can also be categorized into three effective training sections (sections 1 to 3) and one ineffective training section (section 0). Across all types (from Type 1 to Type 12), the starting point *M* and the ending point *N* of the linear trajectory are located on distinct curves of *C*_i_*C*_j_ (where i ≠ j) when the trajectory falls within the effective sections. Conversely, when the trajectory is in the ineffective section, both points *M* and *N* are situated on the same curve of *C*_i_*C*_j_.

Drawing from the 12 distinct types of action spaces, we have distilled a set of universal characteristics and patterns that define the linear trajectories for joint rehabilitation. This comprehensive summary provides a foundational framework for understanding the nuances of effective rehabilitation strategies across various patient scenarios and recovery stages.

In the ineffective section 0, the linear trajectory fails to encompass the joint’s ultimate flexion or its maximum motion range, rendering it ineffective for joint rehabilitation purposes.

In the effective section 1, the linear trajectory exclusively includes the knee joint’s ultimate flexion, which can manifest in two scenarios. If section 0 is positioned at the bottom of the action space, as the linear trajectory ascends, there is an increase in the knee joint’s motion range, the hip joint’s maximum flexion angle, and the hip joint’s motion range, leading to enhanced training intensity. Conversely, when section 0 is at the top or absent, the frequency of the knee joint’s ultimate flexion diminishes under constant speed and time conditions. Thus, the linear trajectory in section 1 primarily targets knee joint rehabilitation with supplementary hip joint involvement.

In the effective section 2, the linear trajectory encompasses three distinct situations. When y*_P_*_14_ > y*_P_*_23_, the trajectory includes both the knee joint’s ultimate flexion and its maximum motion range. Additionally, as the trajectory’s position elevates, both the hip joint’s ultimate flexion and maximum motion range increase. When y*_P_*_14_ < y*_P_*_23_, the trajectory includes the hip joint’s ultimate flexion and maximum motion range, with a concurrent increase in the knee joint’s maximum flexion angle and maximum motion range as the trajectory rises. When y*_P_*_14_ = y*_P_*_23_, the effective section 2 can be considered a singular line with *P*_14_ as the starting point and *P*_23_ as the end point, encompassing the ultimate flexion and motion range of both joints.

Lastly, in the effective section 3, the trajectory solely includes the hip joint’s ultimate flexion, presenting two scenarios. If section 0 is at the bottom or absent, the frequency of the hip joint’s ultimate flexion decreases as the trajectory’s position increases under the same speed and time. When section 0 is at the top, the hip joint’s motion range, the knee joint’s maximum flexion angle, and the knee joint’s motion range all increase, leading to more intensive training. Consequently, the linear trajectory in section 3 is primarily focused on hip joint rehabilitation with supplementary knee joint involvement.

As shown in Table 3, the influence of the linear trajectory’s sectional division on joint rehabilitation is universally applicable across all types, ensuring a tailored approach to accommodate the varying needs of patients. This systematic categorization into effective and ineffective training sections allows for precise calibration of the rehabilitation process, optimizing the trajectory’s design to enhance joint recovery and function. By leveraging this comprehensive framework, clinicians can effectively tailor rehabilitation programs to achieve the best possible outcomes for patients at different stages of recovery.

### 3.3. Generation of Rehabilitation Path

**Figure 12:**
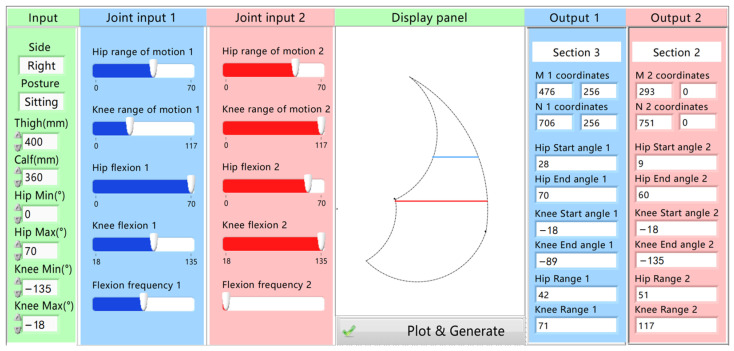
Operating system for generating linear rehabilitation paths based on joint rehabilitation indicators.

It is challenging for physicians to directly plan rehabilitative trajectories based on the joint rehabilitation needs they design because they cannot intuitively plan matching trajectories within the action space. Therefore, this paper proposed a rehabilitation system that enables physicians to plan trajectories based on joint training indicators according to the types of patient’s action space, as illustrated in Figure 12. Moreover, the action space and the coordinates of the linear trajectory on the display panel are obtained based on Equation (3). Five joint rehabilitation indicators were considered and provided on the control panel for physician adjustment. These include the range of motion of the hip joint, the range of motion of the knee joint, the degree of hip joint flexion, the degree of knee joint flexion, and flexion frequency. Physicians customize five joint training parameters for the patient’s rehabilitation by adjusting the five corresponding controls on the operation panel. Subsequently, the system generates the corresponding trajectory based on the joint rehabilitation requirements set by the physician.

Once the physician enters the lower-limb parameters of the patient in the first column, including leg length and joint mobility, the system generates a corresponding action space on the board. Concurrently, the joint rehabilitation indicator adjustment controls are designed to update in real time to match the patient’s current joint mobility. Recognizing that it is challenging for physicians to plan corresponding rehabilitation paths on the user interface based on the formulated joint rehabilitative requirements, the system incorporates the relationship between joint rehabilitation indicators and trajectories. This is to generate a matching rehabilitation path, as analyzed earlier. By setting five joint rehabilitation indicators in the second column, the system automatically generates a matching trajectory, assisting the physician in creating a straight-line trajectory that matches the setting joint rehabilitation needs. Both the motion space and the straight trajectory are depicted in the third column. Finally, the panel displays the maximum and minimum flexion angles of the joints, the range of motion of the joint, and the interval where the straight-line trajectory lies. This comprehensive integration of physiological inputs and system-generated trajectories allows for a more effective and customized joint rehabilitation plan tailored to the patient’s specific requirements and facilitates the rehabilitation process.

## 4. Rehabilitative Trajectory Planning-Based Jerk Minimization

### 4.1. Polynomial Trajectory Planning

**Figure 13:**
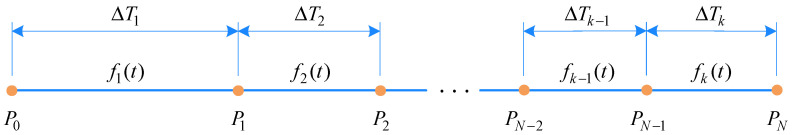
The trajectory composed of multiple segments.

Based on the initial and final position of the rehabilitation trajectory generated by the joint rehabilitative indicators, different paths with different motion parameters by the robot end effector can be generated [41]. It is common to split the trajectory into multiple segments, as shown in Figure 13. There are N+1 key points $P_{i}(0\leq i\leq N)$ on the trajectory to connect the adjacent segments. The rehabilitation trajectory can be represented by polynomials with *k*-segments. Every single segment can be expressed by a polynomial of degree *n*, written as

(7) $$f(t)=p_{0}+p_{1}t+p_{2}t^{2}+\cdots +p_{n}t^{n}.$$

Through writing the *n*th-order coefficients in a vector form $p={\left[\begin{array}{cccc} p_{0} & p_{1} & \cdots & p_{n} \end{array}\right]}^{⊤}$, and then differentiating them, the trajectory of a single segment, as well as the velocity, acceleration, and the jerk can be represented as

(8) $$\begin{array}{l} p(t)=f(t)=\left[\begin{array}{llllllll} 1 & t & t^{2} & t^{3} & t^{4} & t^{5} & \cdots & t^{n} \end{array}\right]p=Pp\\ v(t)=f^{\prime }(t)=\left[\begin{array}{llllllll} 0 & 1 & 2t & 3t^{2} & 4t^{3} & 5t^{4} & \cdots & nt^{n-1} \end{array}\right]p=Vp\\ a(t)=f^{\prime \prime }(t)=\left[\begin{array}{llllllll} 0 & 0 & 2 & 6t & 12t^{2} & 20t^{3} & \cdots & \frac{n!}{(n-2)!}t^{n-2} \end{array}\right]p=Ap\\ j(t)=f^{\prime \prime \prime }(t)=\left[\begin{array}{llllllll} 0 & 0 & 0 & 6 & 24t & 60t^{2} & \cdots & \frac{n!}{(n-3)!}t^{n-3} \end{array}\right]p=Jp \end{array}.$$

The whole trajectory in Figure 13 is separated into multiple segments connected by the key points, and it can be expressed as a multi-segment trajectory. The *i*-th segment is written as

(9) $$\begin{array}{ccc} f_{i}(t)=\left[\begin{array}{lllll} 1 & t & t^{2} & \cdots & t^{n} \end{array}\right]p_{i}, & t_{i-1}\leq t<t_{i}, & i=1,2, \end{array}\cdots ,k$$

where $t_{i}-t_{i-1}$ is the execution time, which is equal to the proportion of the length of the *i*-th segment, *k* is the number of segments, and $p_{i}={\left[\begin{array}{cccc} p_{i0} & p_{i1} & \cdots & p_{in} \end{array}\right]}^{⊤}$ is the parameters of polynomials of the *k*-th segment.

### 4.2. Trajectory Optimization

In traditional rehabilitation therapies, physicians commonly drag the patient’s foot to complete the end linear motion of flexion–extension of the lower limbs. During this process, they provide small shock, smooth, and stable rehabilitation trajectories through manual techniques and experience. To reduce the impact and vibration of lower-limb rehabilitation robots and track smoother and more stable trajectories, the impact along the trajectory will be minimized, and a target function represented in a quadratic form is constructed as

(10) $$\begin{array}{cc} \min & j_{i}^{2}(t)={\left(f^{\prime \prime \prime }(t)\right)}^{2} \end{array}.$$

By combining the objective function with Equation (9), we can obtain

(11) ∫0T(f‴(t))2dt=∑i=1k∫ti−1ti(f(3)(t))2dt=∑i=1k∫ti−1ti(Jip)⊤(Jip)dt=∑i=1kpT∫ti−1tiJiJi⊤dt⋅p=∑i=1kpTQip.

In the equation, $Q_{i}$ is a block matrix, and it can be expressed as

(12) $$Q_{i}=\int_{t_{i-1}}^{t_{i}}J^{T}Jdt=\left[\begin{array}{cc} 0_{3\times 3} & 0_{3\times (n-2)}\\ 0_{(n-2)\times 3} & \frac{\left(r-1)(r-2)(r-3)(c-1)(c-2)(c-3\right)}{\left(r+c-7\right)}(t_{i}^{r+c-7}-t_{i-1}^{r+c-7}) \end{array}\right],$$

where *r* and *c* stand for the row and column indices of the matrix, respectively. It is seen that the fourth block of $Q_{i}$ is a none-zero entry, which is expressed in the form of a diagonal matrix:

(13) $$Q=diag\left[\begin{array}{cccc} Q_{1} & Q_{2} & \cdot \cdot \cdot & Q_{k} \end{array}\right].$$

In traditional rehabilitation therapies, physicians tend to impose certain limitations on the starting and ending points of the rehabilitation trajectory as well as ensure the continuity of the trajectory. Thus, assuming that at the initial point in the first segment of the trajectory, the initial position, velocity, and acceleration equal to $p_{0}$, $v_{0}$, and $a_{0}$, respectively, the corresponding constraints can be set to

(14) $$\begin{array}{l} p(t_{0})=\left[\begin{array}{cccccccc} 1 & t_{0} & t_{0}^{2} & t_{0}^{3} & t_{0}^{4} & t_{0}^{5} & \cdots & t_{0}^{n} \end{array}\right]p_{1}=P_{0}p_{1}=p_{0}\\ v(t_{0})=\left[\begin{array}{llllllll} 0 & 1 & 2t_{0} & 3t_{0}^{2} & 4t_{0}^{3} & 5t_{0}^{4} & \cdots & nt_{0}^{n-1} \end{array}\right]p_{1}=V_{0}p_{1}=v_{0}\\ a(t_{0})=\left[\begin{array}{llllllll} 0 & 0 & 2 & 6t_{0} & 12t_{0}^{2} & 20t_{0}^{3} & \cdots & n(n-1)t_{0}^{n-2} \end{array}\right]p_{1}=A_{0}p_{1}=a_{0} \end{array}.$$

Similarly, for the ending point of the last segment of the trajectory, the position, velocity, and acceleration constraints can also be specified. To ensure the continuity of the trajectory, the position, velocity, and acceleration of the end point of the current segment are set to be equal to those of the starting point of the next segment. This approach allows for a seamless transition from one movement phase to the next, facilitating smooth and effective rehabilitation processes. The constraint equations for the intersection of two adjacent trajectories can be represented as

(15) $$\begin{array}{l} p_{j}(t_{i})=P_{i}p_{j}=P_{i}p_{j+1}=p_{j+1}(t_{i})\\ v_{j}(t_{i})=V_{i}p_{j}=V_{i}p_{j+1}=v_{j+1}(t_{i})\\ a_{j}(t_{i})=A_{i}p_{j}=A_{i}p_{j+1}=a_{j+1}(t_{i}) \end{array}.$$

The kinematic constraint equations of all the key points mentioned above can be transformed into a huge overarching matrix, which is represented as

(16) $$\left[\begin{array}{c} 1,t_{0},t_{0}^{2},\cdots ,t_{0}^{n},0_{1\times (k-1)(n+1)}\\ 0,1,2t_{0},\cdots ,nt_{0}^{n-1},0_{1\times (k-1)(n+1)}\\ 0,0,2,\cdots ,n(n-1)t_{0}^{n-2},0_{1\times (k-1)(n+1)}\\ \vdots \\ 0_{1\times (i-1)(n+1)},1,t_{i},t_{i}^{2},\cdots t_{i}^{n},0_{1\times (k-i)(n+1)}\\ \vdots \\ 0_{1\times (k-1)(n+1)},1,t_{k},t_{k}^{2},\cdots ,t_{k}^{n}\\ 0_{1\times (k-1)(n+1)},0,1,2t_{k},\cdots ,nt_{k}^{n-1}\\ 0_{1\times (k-1)(n+1)},0,0,2,\cdots ,n(n-1)t_{k}^{n-2}\\ \vdots \\ 0_{1\times (i-1)(n+1)},1,t_{i},t_{i}^{2},\cdots ,t_{i}^{n},-1,-t_{i},-t_{i}^{2},\cdots ,-t_{i}^{n},0_{1\times (k-i-1)(n+1)}\\ 0_{1\times (i-1)(n+1)},0,1,2t_{i},\cdots ,nt_{i}^{n-1},0,-1,-2t_{i},\cdots ,-nt_{i}^{n-1},0_{1\times (k-i-1)(n+1)}\\ 0_{1\times (i-1)(n+1)},0,0,2,\cdots ,n(n-1)t_{i}^{n-2},0,0,-2,\cdots ,-n(n-1)t_{i}^{n-2},0_{1\times (k-i-1)(n+1)}\\ \vdots \end{array}\right]\cdot p=\left[\begin{array}{c} p_{0}\\ v_{0}\\ a_{0}\\ \vdots \\ p_{i}\\ \vdots \\ p_{N}\\ v_{N}\\ a_{N}\\ \vdots \\ 0\\ 0\\ 0\\ \vdots \end{array}\right].$$

Based on Equations (11) and (13), the problem of solving the jerk is simplified as a quadratic programming problem: $\overset{k}{\underset{i=1}{\sum }}j_{i}=p^{T}Qp$. Among them, vector *p* represents the sum of *n*-th degree polynomial coefficients of *k* trajectory segments, including k*(*n* + 1) coefficients to be solved. *Q* represents the summary of the matrix *Q_i_* for each trajectory segment, which is a symmetric matrix considered the Hessian matrix for the quadratic programming problems. According to Equation (12), since *r* ≥ 4, *c* ≥ 4, and *t_i_* > *t_i_*_−1_, it can be determined that *Q* is a semi-positive definite matrix. Therefore, the problem of solving the minimum jerk can be regarded as a convex quadratic programming problem, and there exists a global optimal solution.

On this basis, an optimization function $quadprog(Q,p,[],[],A_{eq},b_{eq})$ was used in MATLAB R2022a to solve quadratic programming problems, where *Q* is the Hessian matrix in quadratic programming, corresponding to Equation (13). *p* represents the summary of *n*-th degree polynomial coefficients for *k* trajectory segments. The coefficient matrix and the right-hand vector of the linear inequality constraint represented by items 3 and 4 do not exist and are therefore set to be empty. *A_eq_* represents the coefficient matrix of linear equality constraints, corresponding to the large matrix of the first term on the left side of Equation (16). *b_eq_* represents the right-hand vector of the linear equality constraint, corresponding to the vector on the right-hand side of Equation (16). Finally, based on the equality constraint conditions and the quadratic term matrix, the coefficient vector of the first-order term in the quadratic programming that minimizes the total jerk can be obtained, which is the *n*-th degree polynomial coefficient of each trajectory segment.

### 4.3. Generation of Rehabilitation Trajectory

**Figure 14:**
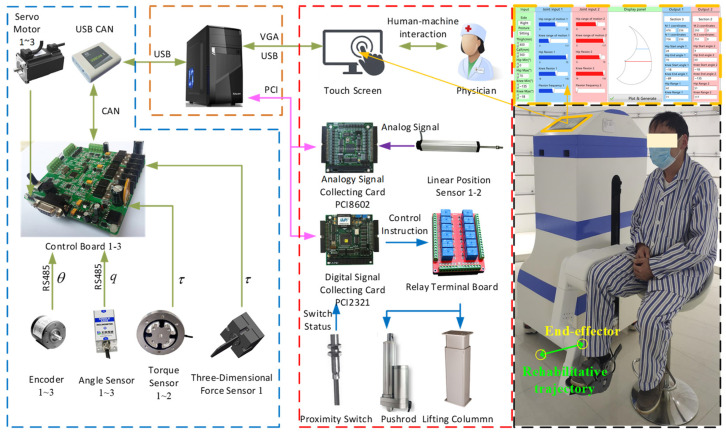
Hardware system and experimental platform for trajectory tracking.

**Figure 15:**
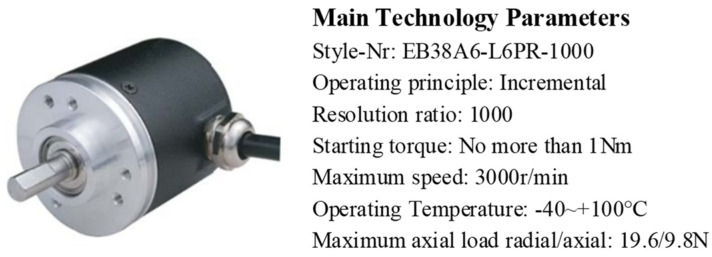
Main technology parameters of the encoder.

As shown in Figure 14, the jerk minimization trajectory planning is applied to the rehabilitation robot system, which is controlled using the LabView software (NI LabVIEW 2018). In the experiment, the motion information of the robot joints, such as joint displacement and velocity, is read from the encoder of the motor, with its main technology parameters shown in Figure 15. Furthermore, the acceleration and jerk can be obtained by performing once and twice differentiations on the encoder’s angular velocity, respectively. Multiple repeated experiments were conducted to calculate the average value, aiming to minimize the deviations. The motion of the end effector is calculated through forward kinematics.

Based on the rehabilitation needs for joints, the initial position is set at the end point of the linear trajectory located far from the hip joint. Analogous to the traditional therapeutic approach where physicians drag the affected limb, a complete cycle of linear rehabilitation exercise involves a single flexion and extension of the lower limb, corresponding to the robot’s end effector moving from point *N* to point *M* and then returning to point *N*. The coordinates of key points of the two trajectories in Figure 12 are shown in Table 3. Subsequently, the linear rehabilitation trajectory is composed of 14 segments, and a seventh-order polynomial is used from the perspective of impact continuity and time consumption. Then, the Cartesian coordinate representation of each segment is expressed as

(17) $$\left\{x_{i}(t_{i}),y_{i}(t_{i})\right\}=p_{0}+p_{1}t_{i}+p_{2}t_{i}^{2}+p_{3}t_{i}^{3}+p_{4}t_{i}^{4}+p_{5}t_{i}^{5}+p_{6}t_{i}^{6}+p_{7}t_{i}^{7}$$

It is set that the distance between key points is equal. The estimated execution time for the robot’s operation is set to TE_1 = 8.4 s and TE_2 = 16.8 s, respectively, based on the proportional relationship between the lengths of the two trajectories. The trajectory optimization problem is solved using the MATLAB quadprog optimization algorithm (the quadratic programming solver). The random trajectories are also generated using a seventh-order polynomial method, with constraints set on the initial and terminal points of the trajectory, as well as the first and last points of adjacent trajectories. However, the minimization of jerk is not the objective, which implies that it does not involve quadratic programming problems. Table 4 represents the coordinates of the key points. All trajectories pass through the same key points and can both achieve linear rehabilitative trajectories, but there are differences in the outlines.

**Figure 16:**
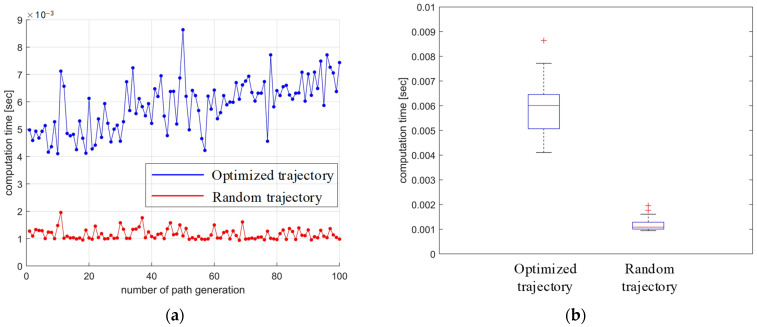
The computational time of trajectory generation: (**a**) line chart; (**b**) box chart.

The comparison of the computational time between the optimized and randomly generated trajectories is shown in Figure 16. Mostly, the computational time of the optimized trajectory is about three times higher than the one without optimization. Despite all this, the computation time of optimization is short enough to be accepted in real robot rehabilitation.

**Figure 17:**
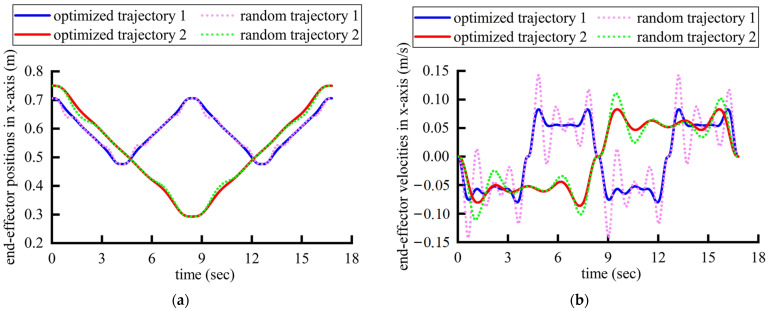

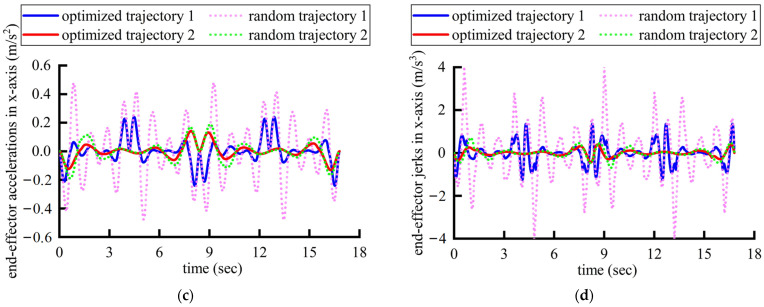
The motion data of the robot’s end effector in Cartesian space: (**a**) positions; (**b**) velocities; (**c**) accelerations; (**d**) jerks.

Figure 17 shows the position, velocities, accelerations, and jerks of the robot’s end effector in Cartesian space, which means that the optimized trajectories can ensure smaller and more continuous jerks compared with the randomly generated ones. Besides differing from the random trajectories, it conforms more closely to the trajectory characteristics during traditional rehabilitation trajectories by the physicians dragging the affected limb. The corresponding polynomial coefficients and execution times for the optimized trajectories in each segment on the x-axis are shown in Table 5. Evidently, the two rehabilitation paths generated based on two joint rehabilitative indicators have distinct end effector displacement. It is relatively challenging for physicians to accurately plan the matching rehabilitation path solely based on the set of joint rehabilitative indicators.

**Figure 18:**
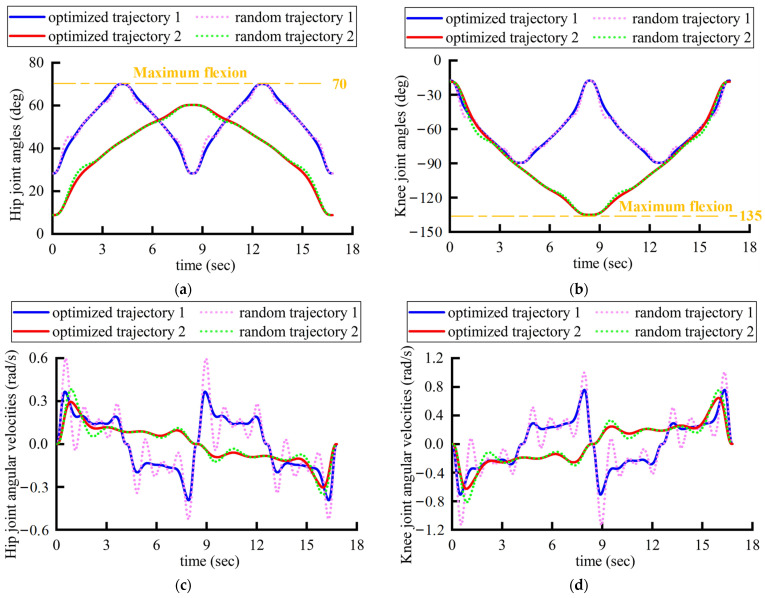

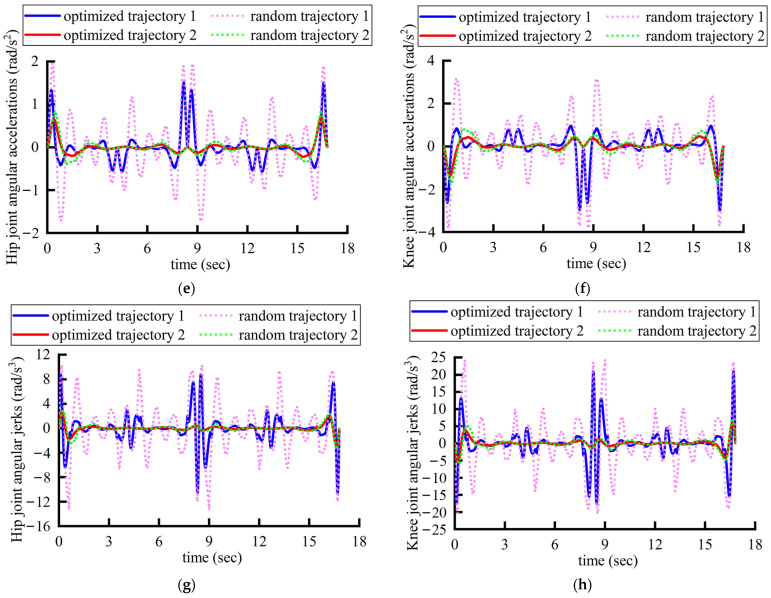
The motion data of the robot’s joints for the optimized trajectories and random trajectories: (**a**) displacements of hip; (**b**) displacements of knee; (**c**) velocities of hip; (**d**) velocities of knee; (**e**) accelerations of hip; (**f**) accelerations of knee; (**g**) jerks of hip; (**h**) jerks of knee.

The discrete coordinates of several points on the robot’s end effector trajectory, which have been optimized from the previous section, are subjected to the kinematic inverse solution. This yields the angular displacements at the hip and knee joints corresponding to these points. Subsequently, these angular displacements are input into the motor controller, where they are processed through a PD control algorithm to achieve the rehabilitation trajectory planned earlier. The angular displacements, angular velocities, angular accelerations, and jerks for the hip and knee joints are shown in Figure 18. It can be observed that the joint angular velocity and angular acceleration curves achieved through the seventh-order polynomial planning are both smooth and continuous. The jerk curves show that the starting and the ending positions of the joint exhibit zero jerk, aligning well with the lower-limb rehabilitation requirements of the patient.

A comparison reveals that the optimized trajectories’ joint velocities, accelerations, and jerks are generally of a lower magnitude than those of a randomly generated trajectory. It is noted that all the maximum magnitudes of the joint velocity, acceleration, and jerk profiles of the optimized trajectory are smaller than the random ones, which implies that the optimized trajectory ensures a higher kinematic performance of the robot’s rehabilitation training. This implies that the optimized one better conforms to the kinematic characteristics of traditional rehabilitation therapies, where a physician manually drags the affected limbs. The optimized trajectories ensure lighter impacts and a smoother path, thereby guaranteeing the robot with higher motional performance in rehabilitation.

Additionally, as shown in Figure 18a,b, a comparison of the two rehabilitation trajectories reveals that trajectory 1 completes one training cycle within 16.8 s, with the knee joint reaching its maximum flexion at the 8.4-th second, and all ranges of motion meet the set joint rehabilitative criteria. Trajectory 2 completes two training cycles within 16.8 s, with the hip joint reaching its maximum flexion at the 4.2-th second and at the 12.6-th second, with all movement ranges also meeting the set joint rehabilitative criteria. Moreover, the joint velocity and acceleration curves are smooth and continuously differentiable, and the joint jerk is restricted to zero at the start point, end point, and the point of the maximum flexion, effectively replicating the rehabilitation patterns that occur during traditional rehabilitation therapy, where a physician drags the affected limb. This approach maximizes the safety of the patient’s rehabilitation training and simulates the reliable technique of the physician.

As shown in Figure 18g,h, the maximum hip joint jerk of the random trajectory 1 is 10.2 rad/s^3^, and the maximum knee joint impact is 24.9 rad/s^3^. The maximum jerk on the hip joint of optimized trajectory 1 is 9.3 rad/s^3^, and the maximum jerk on the knee joint is 21.3 rad/s^3^. The maximum jerk on the hip joint of random trajectory 2 is 2.9 rad/s^3^, and the maximum jerk on the knee joint is approximately 5.8 rad/s^3^. The maximum jerk on the hip joint of trajectory 1 after optimization is 2.5 rad/s^3^, and the maximum jerk on the knee joint is 5.5 rad/s^3^. Therefore, the maximum joint jerk of optimized trajectories 1 and 2 is smaller than that of the random trajectories. It is vital in rehabilitation because the maximum jerk is the most likely to cause secondary damage, and the optimized trajectory effectively reduces the maximum jerk during training. In addition, the joint jerk at each moment of the optimized trajectory is almost always smaller than that of the random trajectory, indicating that patients can feel smaller impacts during the overall rehabilitative process, which helps with the comfort of rehabilitation. In summary, the optimized trajectory can effectively improve the safety and comfort of patients’ lower-limb joint rehabilitation compared to the random trajectory.

## 5. Conclusions

This paper introduces a novel linear rehabilitative motion planning approach and successfully demonstrates its integration with a three-degrees-of-freedom (DOF) multi-posture lower-limb rehabilitation robot. Addressing the challenge of incorporating the nuanced treatment expertise of physicians into robotic rehabilitation, we have analyzed the interplay between joint rehabilitation indicators and linear rehabilitation trajectories across diverse action spaces. Subsequently, we designed a joint rehabilitation indicator-based path generation system to enhance the efficiency of rehabilitation path planning for clinicians using the lower-limb rehabilitation robot. To ensure the continuity and minimize the jerks of rehabilitation trajectories, we employed high-order polynomial curves in the trajectory planning process. This approach was further refined through the resolution of a constrained quadratic optimization problem, effectively reducing the jerk of the end effector and the joints of the robot.

Through comparative studies involving Cartesian trajectories determined at equivalent key points, the results revealed that trajectory optimization is indeed feasible for real-time trajectory planning, as evidenced by the low computation times (not exceeding 10 ms). The optimization of trajectories using polynomial curves was found to significantly outperform random trajectories in enhancing the robot’s rehabilitative training performance, thereby validating the efficacy of our method within the rehabilitation technology. Furthermore, a comparison of two sets of optimized rehabilitation trajectories, one before and one after the application of our method, confirmed that the robot’s performance was notably improved. Specifically, trajectory 1 and trajectory 2 were tailored to meet the physician’s requirements for hip joint extreme flexion and knee joint extreme flexion, respectively. Additionally, both optimized trajectories adhered to other predefined joint rehabilitation indicators, substantiating the effectiveness of our joint rehabilitation indicator-based path generation system in assisting physicians in the operation of rehabilitation robots.

## Figures and Tables

**Table 1:** The maximum joint ranges of motion of MHLRR under different postures.

Joint/Posture	Lying	Sitting	Standing
Hip	0°~130°	0°~80°	−20°~60°
Knee	−135°~0°	−135°~0°	−135°~0°
Ankle	−45°~30°	−45°~30°	−45°~30°

**Table 2:** The 12 types of action spaces based on the classification method.

Type	Section Numbers	Combination of Figure 6, Figure 7 and Figure 8	Demarcation Point and Arc Pair (from Bottom to Top)	Discriminant Condition (*y_P_*_14_ − *y_P_*_23_ = *l*_1_∙sin*α*_max_ − *l*_1_∙sin*α*_min_+ *l*_2_∙sin(*α*_max_ + *β*_min_) − *l*_2_∙sin(*α*_min_ + *β*_max_))	
1	3	Figure 6a, Figure 7a and Figure 8a Figure 6c, Figure 7d and Figure 8d	*P*_34_-*P*_23_-*P*_14_-*P*_12_ *C*_4_*C*_3_-*C*_4_*C*_2_-*C*_1_*C*_2_	*y_P_*_14_ − *y_P_*_23_ > 0	(a). *α*_min_ = 50°; 50° < *α*_max_ ≤ 90°; $(b).\;\alpha_{\min}=50{}^{\circ};\;90{}^{\circ}\;<\;\alpha_{\max}\;\leq \;130{}^{\circ};\;\beta_{\max}\;\geq \;180{}^{\circ};\;\beta_{\max}\geq 180{}^{\circ}-\alpha_{\max}-\arccos(\frac{l_{1}}{l_{2}}\cdot \cos\alpha_{\max})$ (c). *α*_min_ = 0°; −90° ≤ *β*_min_ < 0°; Condition (a) and (b) are in lying position. Condition (c) is in sitting position.
2	3	Figure 6a, Figure 7b and Figure 8a Figure 6c, Figure 7e and Figure 8d	*P*_34_-*P*_14_-*P*_23_-*P*_12_ *C*_4_*C*_3_-*C*_1_*C*_3_-*C*_1_*C*_2_	*y_P_*_14_ − *y_P_*_23_ < 0	
3	2	Figure 6a, Figure 7c and Figure 8a Figure 6c, Figure 7f and Figure 8d	*P*_34_-*P*_14_(*P*_23_)-*P*_12_ *C*_4_*C*_3_-*C*_1_*C*_2_	*y_P_*_14_ − *y_P_*_23_ = 0	
4	4	Figure 6a, Figure 7a and Figure 8b	*P*_34_-*P*_23_-*P*_14_-*P*_12_-*Q*_2_ *C*_4_*C*_3_-*C*_4_*C*_2_-*C*_1_*C*_2_-*C*_2_*C*_2_	*y_P_*_14_ − *y_P_*_23_ > 0	(d). *α*_min_ = 50°; 50° < *α*_max_ ≤ 90°; −*α*_max_ − 85° ≤ *β*_max_ ≤ −*α*_max_ + 90°; $\beta_{\max}\geq 180{}^{\circ}-\alpha_{\max}-\arccos(\frac{l_{1}}{l_{2}}\cdot \cos\alpha_{\max})$; Condition (d) is in lying position.
5	4	Figure 6a, Figure 7b and Figure 8b	*P*_34_-*P*_14_-*P*_23_-*P*_12_-*Q*_2_ *C*_4_*C*_3_-*C*_1_*C*_3_-*C*_1_*C*_2_-*C*_2_*C*_2_	*y_P_*_14_ − *y_P_*_23_ < 0	
6	3	Figure 6a, Figure 7c and Figure 8b	*P*_34_-*P*_14_(*P*_23_)-*P*_12_-*Q*_2_ *C*_4_*C*_3_-*C*_1_*C*_2_-*C*_2_*C*_2_	*y_P_*_14_ − *y_P_*_23_ = 0	
7	4	Figure 6a, Figure 7a and Figure 8c	*P*_34_-*P*_23_-*P*_14_-*Q*_1_-*Q*_2_ *C*_4_*C*_3_-*C*_4_*C*_2_-*C*_1_*C*_2_-*C*_2_*C*_2_	*y_P_*_14_ − *y_P_*_23_ > 0	(e). *α*_min_ = 50°; 90° < *α*_max_ ≤ 130°; −*α*_max_ − 85° ≤ *β*_max_ ≤ −*α*_max_ + 90°; $\beta_{\max}\geq 180{}^{\circ}-\alpha_{\max}-\arccos(\frac{l_{1}}{l_{2}}\cdot \cos\alpha_{\max})$; Condition (e) is in lying position.
8	4	Figure 6a, Figure 7b and Figure 8c	*P*_34_-*P*_14_-*P*_23_-*Q*_1_-*Q*_2_ *C*_4_*C*_3_-*C*_1_*C*_3_-*C*_1_*C*_2_-*C*_2_*C*_2_	*y_P_*_14_ − *y_P_*_23_ < 0	
9	3	Figure 6a, Figure 7c and Figure 8c	*P*_34_-*P*_14_(*P*_23_)-*Q*_1_-*Q*_2_ *C*_4_*C*_3_-*C*_1_*C*_2_-*C*_2_*C*_2_	*y_P_*_14_ − *y_P_*_23_ = 0	
10	4	Figure 6b, Figure 7d and Figure 8d	*Q*_3_-*P*_34_-*P*_23_-*P*_14_-*P*_12_ *C*_3_*C*_3_-*C*_4_*C*_3_-*C*_4_*C*_2_-*C*_1_*C*_2_	*y_P_*_14_ − *y_P_*_23_ > 0	(f). *α*_min_ = 0°; −135° ≤ *β*_min_ < −90°; Condition (f) is in sitting position.
11	4	Figure 6b, Figure 7e and Figure 8d	*Q*_3_-*P*_34_-*P*_14_-*P*_23_-*P*_12_ *C*_3_*C*_3_-*C*_4_*C*_3_-*C*_1_*C*_3_-*C*_1_*C*_2_	*y_P_*_14_ − *y_P_*_23_ < 0	
12	3	Figure 6b, Figure 7f and Figure 8d	*Q*_3_-*P*_34_-*P*_14_(*P*_23_)-*P*_12_ *C*_3_*C*_3_-*C*_4_*C*_3_-*C*_1_*C*_2_	*y_P_*_14_ − *y_P_*_23_ = 0	

Figure 6, Figure 7, and Figure 8, respectively, represent the various distributions of key points in the lower, middle, and upper regions. The table lists 12 types of action spaces, which are combinations included in Figure 6, Figure 7 and Figure 8. The third column enumerates the combination cases corresponding to each type.

**Table 3:** The relationship between joint rehabilitation requirements and the positions of linear trajectories applicable to all types of action spaces.

Type 1~12	Primary Indicators of Joint Rehabilitation		Auxiliary Indicators of Joint Rehabilitation	
	Relationship of *P*_14_ and *P*_23_	Change Rules as Position Rises	Position of Section 0	Change Rules as Position Rises
section 0	/	/	/	/
section 1	All is permissible	*β_L_*_min_ = *β*_min_	At top or not exist	Hip flexion frequency increases.
			At bottom	*α_L_*_max_, *α_L_*_min_ and *β_L_*_max_ increase.
section 2	*y_P_*_14_ > *y_P_*_23_	*β_L_*_max_ = *β*_max_; *β_L_*_min_ = *β*_min_	All is permissible	*α_L_*_max_ and *α_L_*_min_ increase.
	*y_P_*_14_ < *y_P_*_23_	*α_L_*_max_ = *α*_max_; *α_L_*_min_ = *α*_min_		*β_L_*_max_ and *β_L_*_min_ increase.
	*y_P_*_14_ = *y_P_*_23_	*α_L_*_max_ = *α*_max_; *α_L_*_min_ = *α*_min_; *β_L_*_max_ = *β*_max_; *β_L_*_min_ = *β*_min_		Not exist
section 3	All is permissible	*α_L_*_max_ = *α*_max_	At top or not exist	*α_L_*_min_, *β_L_*_max_ and *β_L_*_min_ increase.
			At bottom	Knee flexion frequency increases.

It is a summary of the relationship between the joint rehabilitation characteristics and the positions of linear trajectories under all 12 types of action spaces. This allows physicians to quickly identify the specific section and the position of the corresponding linear trajectory based on the set joint rehabilitative requirements.

**Table 4:** The key points for the two trajectories of the rehabilitation robot.

No.	0	1	2	3	4	5	6	7	8	9	10	11	12	13	14
Pi(1)	$\left[\begin{array}{c} 0.71\\ 0.26 \end{array}\right]$	$\left[\begin{array}{c} 0.67\\ 0.26 \end{array}\right]$	$\left[\begin{array}{c} 0.64\\ 0.26 \end{array}\right]$	$\left[\begin{array}{c} 0.61\\ 0.26 \end{array}\right]$	$\left[\begin{array}{c} 0.57\\ 0.26 \end{array}\right]$	$\left[\begin{array}{c} 0.54\\ 0.26 \end{array}\right]$	$\left[\begin{array}{c} 0.51\\ 0.26 \end{array}\right]$	$\left[\begin{array}{c} 0.48\\ 0.26 \end{array}\right]$	$\left[\begin{array}{c} 0.51\\ 0.26 \end{array}\right]$	$\left[\begin{array}{c} 0.54\\ 0.26 \end{array}\right]$	$\left[\begin{array}{c} 0.57\\ 0.26 \end{array}\right]$	$\left[\begin{array}{c} 0.61\\ 0.26 \end{array}\right]$	$\left[\begin{array}{c} 0.64\\ 0.26 \end{array}\right]$	$\left[\begin{array}{c} 0.67\\ 0.26 \end{array}\right]$	$\left[\begin{array}{c} 0.71\\ 0.26 \end{array}\right]$
Pi(2)	$\left[\begin{array}{c} 0.75\\ 0 \end{array}\right]$	$\left[\begin{array}{c} 0.69\\ 0 \end{array}\right]$	$\left[\begin{array}{c} 0.62\\ 0 \end{array}\right]$	$\left[\begin{array}{c} 0.55\\ 0 \end{array}\right]$	$\left[\begin{array}{c} 0.49\\ 0 \end{array}\right]$	$\left[\begin{array}{c} 0.42\\ 0 \end{array}\right]$	$\left[\begin{array}{c} 0.36\\ 0 \end{array}\right]$	$\left[\begin{array}{c} 0.29\\ 0 \end{array}\right]$	$\left[\begin{array}{c} 0.36\\ 0 \end{array}\right]$	$\left[\begin{array}{c} 0.42\\ 0 \end{array}\right]$	$\left[\begin{array}{c} 0.49\\ 0 \end{array}\right]$	$\left[\begin{array}{c} 0.55\\ 0 \end{array}\right]$	$\left[\begin{array}{c} 0.62\\ 0 \end{array}\right]$	$\left[\begin{array}{c} 0.69\\ 0 \end{array}\right]$	$\left[\begin{array}{c} 0.75\\ 0 \end{array}\right]$

The units of coordinates are all in meters.

**Table 5:** Polynomial coefficients and execution time in each segment of the two trajectories.

Segs	$\mathrm{Coefficients}\;\left[\begin{array}{llllllll} p_{0}, & p_{1}, & p_{2}, & p_{3}, & p_{4}, & p_{5}, & p_{6}, & p_{7} \end{array}\right]$	*t_i_* (s)
1	p1(1_x):0.7500000−1.91034.7426−4.40701.4838	0.70
	p1(2_x):0.7500000−0.16320.2185−0.10930.0198	1.35
2	p2(1_x):0.6847−0.14690.03890.1942−0.75881.3593−1.46020.6930	0.50
	p2(2_x):0.6847−0.07780.01450.0325−0.05780.0474−0.02390.0054	1.15
3	p3(1_x):0.6194−0.1165−0.0270−0.02990.11110.0264−0.15800.0765	0.60
	p3(2_x):0.6194−0.0523−0.0086−0.00610.00914.7142e−04−0.00255.7441e−04	1.10
4	p4(1_x):0.5541−0.12210.03160.0241−0.10490.0718−0.01740.0083	0.60
	p4(2_x):0.5541−0.05860.00790.0022−0.00860.0033−3.5354e−048.5121e−05	1.20
5	p5(1_x):0.4889−0.1077−0.0166−0.00650.0605−0.0093−0.08720.0496	0.60
	p5(2_x):0.4889−0.0582−0.00720.00300.0073−0.0015−0.00195.5451e−04	1.10
6	p6(1_x):0.4236−0.1126−0.0035−0.0411−0.27160.9252−1.42620.9456	0.50
	p6(2_x):0.4236−0.04650.0100−0.0088−0.02570.0311−0.01900.0055	1.15
7	p7(1_x):0.3583−0.1576−0.03260.2317−0.71512.8838−4.14111.8915	0.70
	p7(2_x):0.3583−0.0830−0.01800.0417−0.06620.1535−0.12340.0313	1.35
8	p8(1_x):0.29300002.4213−6.38256.3295−2.2830	0.70
	p8(2_x):0.29300000.1844−0.25430.1309−0.0244	1.35
9	p9(1_x):0.35830.1624−0.0356−0.25980.8849−1.70742.0076−1.0076	0.50
	p9(2_x):0.35830.0793−0.0170−0.03620.0619−0.04790.0230−0.0050	1.15
10	p10(1_x):0.42360.1071−0.01130.0564−0.0489−0.05800.1083−0.0455	0.60
	p10(2_x):0.42360.05010.01150.0066−0.0107−2.2438e−040.0027−6.2319e−04	1.10
11	p11(1_x):0.48890.1102−0.0046−0.00850.0375−0.03640.0191−0.0091	0.60
	p11(2_x):0.48890.0591−0.0089−0.00280.0100−0.00383.5664e−04−8.4915e−05	1.20
12	p12(1_x):0.55410.11020.0046−0.0085−0.0178−0.01210.0828−0.0455	0.60
	p12(2_x):0.55410.05910.0089−0.0028−0.00840.00190.0021−6.2319e−04	1.10
13	p13(1_x):0.61940.10710.01130.05640.2633−0.97441.5189−1.0076	0.50
	p13(2_x):0.61940.0501−0.01150.00660.0251−0.02900.0175−0.0050	1.15
14	p14(1_x):0.68470.16240.0356−0.25980.8034−3.29094.8573−2.2830	0.70
	p14(2_x):0.68470.07930.0170−0.03620.0569−0.12900.1000−0.0244	1.35

The coordinates of the linear trajectories remain constant in the y direction with minimal variation, so the polynomial coefficients of each segment tend toward 0 and are not reflected in the table. pi(j_x) represents the polynomial coefficient in the x-direction of the *i*-th segment of the *j*-th trajectory.

## Data Availability

Data are contained within the article.
